# MARITAL TRANSITIONS IN MID AND LATE LIFE

**DOI:** 10.1093/geroni/igad104.0306

**Published:** 2023-12-21

**Authors:** Joan Monin, Jason Newsom

## Abstract

Marital transitions are common in mid and late life, yet little is known about what makes some middle aged and older adults thrive and others experience hardship through these transitions. This symposium will discuss antecedents, consequences, and moderators of adjustment to marital transitions in mid to late life. Monin and colleagues will address antecedents of divorce/separation, presenting results from a case control study showing that later stage dementia is associated with a lower likelihood of divorce/separation, and certain neuropsychiatric symptoms are associated with a greater likelihood of divorce/separation. In terms of consequences of relationship dissolution, Bourassa and colleagues will present findings from a study across a 20-year period of adulthood, showing that people with more breakups in adulthood have more advanced biological age in midlife. Two studies will discuss moderators of associations between relationship transitions and well-being. Carr and Choi will discuss the experiences of childless older adults who become widowed or divorced, and whether they suffer poorer mental health than their peers with children. They will show that childless widowed men and divorced men who lost a child to death have dramatically higher levels of social loneliness, relative to other men. Finally, Scheffer and colleagues will talk about the transition out of dementia spousal caregiving. They will present findings indicating that although caregivers’ emotional well-being and energy improves from moving from being an active caregiver to moving out of the caregiving role, those with lower household income had worse outcomes after caregiving ended.

